# Delayed M50/M100 evoked response component latency in minimally verbal/nonverbal children who have autism spectrum disorder

**DOI:** 10.1186/s13229-019-0283-3

**Published:** 2019-08-15

**Authors:** Timothy P. L. Roberts, Junko Matsuzaki, Lisa Blaskey, Luke Bloy, J. Christopher Edgar, Mina Kim, Matthew Ku, Emily S. Kuschner, David Embick

**Affiliations:** 1 Lurie Family Foundations MEG Imaging Center, Department of Radiology, Children’s Hospital of Philadelphia, 3401 Civic Center Blvd, Philadelphia, PA 19104 USA; 2 Center for Autism Research, Children’s Hospital of Philadelphia, Philadelphia, PA USA; 3 Department of Linguistics, University of Pennsylvania, Philadelphia, PA USA

**Keywords:** Minimally verbal/non-verbal children, Autism spectrum disorder, Magnetoencephalography, M50/M100 responses and language impairment, Autism spectrum disorder, Nonverbal, Auditory cortex

## Abstract

Abnormal auditory neuromagnetic M50 and M100 responses, reflecting primary/secondary auditory cortex processing, have been reported in children who have autism spectrum disorder (ASD). Some studies have reported an association between delays in these responses and language impairment. However, as most prior research has focused on *verbal* individuals with ASD *without* cognitive impairment, rather little is known about neural activity during auditory processing in minimally verbal or nonverbal children who have ASD (ASD-MVNV)—children with little or no speech and often significant cognitive impairment. To understand the neurophysiological mechanisms underlying auditory processing in ASD-MVNV children, magnetoencephalography (MEG) measured M50 and M100 responses arising from left and right superior temporal gyri during tone stimuli in three cohorts: (1) MVNV children who have ASD (ASD-MVNV), (2) verbal children who have ASD and no intellectual disability (ASD-V), and (3) typically developing (TD) children. One hundred and five participants (8–12 years) were included in the final analyses (ASD-MVNV: *n* = 16, 9.85 ± 1.32 years; ASD-V: *n* = 55, 10.64 ± 1.31 years; TD: *n* = 34, 10.18 ± 1.36 years). ASD-MVNV children showed significantly delayed M50 and M100 latencies compared to TD. These delays tended to be greater than the corresponding delays in verbal children with ASD. Across cohorts, delayed latencies were associated with language and communication skills, assessed by the Vineland Adaptive Behavior Scale Communication Domain. Findings suggest that auditory cortex neural activity measures could be dimensional objective indices of language impairment in ASD for either diagnostic (e.g., via threshold or cutoff) or prognostic (considering the continuous variable) use.

## Introduction

Language and/or communication impairment is observed in almost all children who have autism spectrum disorder (ASD) [1]^1^ , with a significant fraction classified as minimally verbal or nonverbal [13, 17–19, 33, 47]. Recent estimates indicate that 25 to 30% of children on the autism spectrum are unable to use verbal language to communicate or are minimally verbal [4]. Brain imaging studies have observed that children who have ASD show prolonged/delayed auditory processing compared to typically developing peers (TD), measured by electroencephalography (EEG) or magnetoencephalography (MEG) [3, 7, 9, 11, 39, 42, 45, 59]. In particular, delayed responses have been observed for auditory response components around 50 ms (MEG: M50, EEG: P50 or P1) and 100 ms (MEG: M100, EEG: N100 or N1), the responses primarily produced by neural activity from primary/association auditory cortex [11, 32, 40, 50].

In an early MEG study, Gage et al. [11] reported on children with ASD (aged 8 to 16 years) and showed delayed M100 latencies to sinusoidal tones. Oram Cardy et al. [34] examined M50 and M100 latencies from children and adult controls, children with ASD, children with Asperger’s syndrome, and children with specific language impairment (SLI) and reported that longer M50 latencies predicted impaired receptive language ability. A prior study implicated maturational changes in auditory pathway white matter as influencing conduction velocity and ultimately M100 latency in typically developing children [41]. However, this was not replicated in a cohort of children with ASD (whose M50 and M100 responses to sinusoidal tones were delayed), leading to the hypothesis that another mechanism (e.g., synaptic transmission) may *also* influence auditory latency delay [45]. Examining left and right superior temporal gyrus (STG) activity, Edgar et al. [8] reported delayed latency of left and right STG M50 and right STG M100 responses in children with ASD aged 6 to 15 years, again implicating maturational abnormalities in the development of primary/secondary auditory areas in children with ASD.

A major limitation of work in this area is that most imaging studies have focused on *verbal* children who have ASD and who do not have significant cognitive impairments. Children who have limited or no speech and those who have intellectual disability are frequently excluded from research given anticipated barriers such as tolerating loud sounds and other sensory experiences associated with magnetic resonance imaging and remaining still during an imaging exam [56]. As a result, research on M50/M100 latency delays and their association with language impairment has not been extended to children who have ASD who develop very little or no spoken language (ASD-MVNV). Consequently, we are unaware of whether findings observed in prior studies (obtained in high functioning ASD) would be replicated in minimally verbal/non-verbal children (i.e., would generalize across the ASD population) or whether they would be exacerbated in the more impaired children (suggesting a dimensional proxy of symptom severity). Generalizability and continuity of findings would play an important role in the debate surrounding consideration of the autism spectrum as a continuous scale vs. clustering minimally verbal/non-verbal children with ASD as a distinct diagnostic entity.

To better understand auditory processing in children on the autism spectrum across a wide range of verbal abilities, the present study used magnetoencephalography (MEG) to measure left and right STG M50 and M100 responses to pure tone stimuli in three cohorts: (1) minimally verbal or nonverbal children who have ASD (ASD-MVNV), (2) verbal children who have ASD and no intellectual disability (ASD-V), and (3) typically developing (TD) children. Recording evoked responses in the ASD-MVNV cohort was facilitated using a combined behavioral and technical strategy, referred to as MEG-PLAN, “the MEG Protocol for Low-language/cognitive Ability Neuroimaging” (MEG-PLAN; [22]) (discussed below). The present study examined the hypotheses that STG M50/M100 latencies would be delayed in ASD-MVNV compared to ASD-V and TD indicating that a more pronounced auditory latency delay is associated with poorer prognosis as well as poorer language ability.

## Methods

### Participants

#### Recruitment and inclusion/exclusion criteria

Participants (aged 8 to 12 years) were recruited from the Children’s Hospital of Philadelphia (CHOP). Participants made two visits to CHOP. During the first visit (2–3 weeks prior to the MEG exam), clinical and diagnostic testing was performed to confirm ASD diagnosis, administer cognitive and language assessments, and ensure that all participants met the study inclusion/exclusion criteria. Clinical assessments were performed by a licensed child psychologist with expertise in ASD. Children with ASD had a prior diagnosis, typically made by an expert clinician in CHOP’s Regional Autism Center or, more rarely, by community providers. Given the extensive clinical evaluations upon which original ASD diagnosis was made, an abbreviated diagnostic battery confirmed the original ASD diagnosis. Diagnostic classification was made using the Autism Diagnostic Observation Schedule (ADOS/ADOS-2; [25, 26]) and parent report on the Social Communication Questionnaire (SCQ) [48]. Dimensional symptom severity indices were obtained from the ADOS/ADOS-2 Calibrated Severity Score metric [14]. The Autism Diagnostic Interview-Revised (ADI-R; [49]) was administered with parents for any participants who entered the study without a formal ASD diagnosis made by an expert clinician (e.g., ASD educational classification only) and for any child with a prior ASD diagnosis for whom a diagnostic discordance existed during the evaluation (e.g., a child who exceeded ADOS/ADOS-2 diagnostic cutoffs but was below SCQ cutoff).

For the ASD-V and TD cohorts, cognitive ability was characterized with the Wechsler Intelligence Scale—Fourth Edition (WISC-IV; [62]), the Wechsler Intelligence Scale—Fifth Edition (WISC-V; [63]), or the Differential Ability Scale—Second Edition (DAS-II, [10]). Based on the study protocol and population included in the study, different IQ tests were used [21]. Psychometrics suggest acceptable correlations (*r* = 0.61–0.84) between these tests, all of which are standardized to an average of 100 and SD of 15. To rule out global cognitive delay in the TD and ASD-V groups, participants were required to score at or above the second percentile (SS > 70) on the nonverbal reasoning composite score of the cognitive assessment administered. For the ASD-MVNV cohort, nonverbal cognitive ability in the ASD-MVNV children was assessed with the Leiter International Performance Scale, Third Edition (Leiter-3; [46]). Given significant spoken language limitations in the ASD-MVNV group, there was not a common assessment of language ability that was valid and appropriate across all three cohorts. Thus, the communication domain score from the Vineland Adaptive Behavior Scale, Second Edition (Vineland-II; [57]), or Vineland Adaptive Behavior Scale, Third Edition (Vineland-III; [58]), a parent-report questionnaire of adaptive behavior skills, was used as a proxy for communication skills. For purposes of brevity throughout the manuscript, “Vineland” will be used as an umbrella term for both editions used in the study.

Inclusion criteria for all participants included males or females 8–12 years old with English as a first language in the family home. For the ASD-MVNV cohort, MVNV status was operationally defined as an expressive vocabulary of fewer than 30 words/phrases used spontaneously and communicatively. Inclusion criteria for the TD children included no significant cognitive impairment (described above) and scoring below the cutoffs for ASD on all domains of the ADOS/ADOS-2 as well on parent questionnaires of ASD symptoms. For the ASD-V cohort, stimulant medications were withheld for at least 24 h prior to each study visit (when possible, and with parental consent). Seven of 71 children (all ASD-V) were prescribed stimulant medications at the time of participation. Data from these participants did not show evidence of forming an outlier cluster in terms of M50 and M100 responses and so these data were retained.

Additional exclusion criteria for all participants included (1) claustrophobia; (2) metallic implanted prosthetic or stimulation device including cardiac pacemakers; (3) excessive metallic dental work including braces, non-removable retainers, or other non-removable metal in the body; (4) nonverbal mental age less than 18 months; (5) history of seizure disorder; (6) known neurological (e.g., cerebral palsy, epilepsy) disorders, severe tics, or severe head trauma; (7) sensory (hearing, visual) impairments (by parent report/medical records); and (8) premature birth (earlier than 34 weeks gestation) or significant birth complications. Known genetic conditions were exclusionary for ASD-V and TD groups but not for ASD-MVNV; however, no genetic conditions were reported for any of the ASD-MVNV individuals included in the present sample.

The study was approved by the CHOP Institutional Review Board, and all participants’ families gave written informed consent. As indicated by institutional policy, where competent to do so, children over the age of seven additionally gave verbal assent, in accordance with the principles of the Declaration of Helsinki.

### Auditory stimuli

Sinusoidal tones (300 ms duration; 10 ms ramps) with a pseudo-randomized 600–2000 ms inter-trial interval were presented using a free field loudspeaker (and thus binaurally) approximately 2 m from the participant at 85 dB SPL using Eprime v1.1 experimental software (Psychology Software Tools Inc., Pittsburgh, PA).

### MEG recording

MEG data were obtained in a magnetically shielded room using a 275-channel whole-cortex CTF magnetometer (CTF MEG, Coquitlam, Canada). In most cases, recordings were made with the participant in a supine position to reduce head motion. In a small number of participants in the ASD-MVNV group, parents indicated a preference for the child to be scanned in a seated upright position. No difference in latency has been found between the recording positions although head position is harder to maintain while seated (and consequently, we might expect more trials to be subsequently excluded during analysis). At the start of the session, three head-position indicator coils were attached to the scalp to provide continuous specification of the position and orientation of the MEG sensors relative to the head [42, 43]. Foam wedges were inserted between the side of each participant’s head and the inside of the MEG dewar to increase participant comfort and ensure that the head remained in the same place in the dewar across recording sessions. To minimize fatigue and encourage an awake state, subjects viewed a silent movie projected on to a screen positioned at a comfortable viewing distance. To aid in the identification of eye-blink activity, the electro-oculogram (EOG, bipolar oblique, upper right and lower left sites) was collected. Electrodes were also attached to the left and right collarbone for electrocardiogram (ECG) recording. EOG, ECG, and MEG signals were digitized at 1200 Hz.

For the ASD-MVNV children, MEG recording was supported by a clinical/behavioral and technical protocol developed by our team—the MEG Protocol for Low-language/cognitive Ability Neuroimaging (MEG-PLAN; [22]). Based on stakeholder feedback, MEG-PLAN was developed as an interdisciplinary protocol to be implemented by a team of clinicians, scientists, and MEG technicians in close consultation with participating families. Clinical and behavioral components focus on using parents as partners, with strategies based on the principles of applied behavior analysis, including systematic desensitization and habituation, differential reinforcement, visual supports, and individual tailoring. MEG-PLAN is implemented in three parts via (1) initial assessment, (2) plan and preparation for the family and team, and (3) in vivo support at the MEG visit. The technical portion of MEG-PLAN includes real-time head motion tracking as well as source modeling via age-matched MRI templates, obviating the need for an individual MRI. MEG-PLAN made it feasible for the often-excluded group of ASD-MVNV children to participate in this neuroimaging study.

### Data analysis

**Fig. 1:**
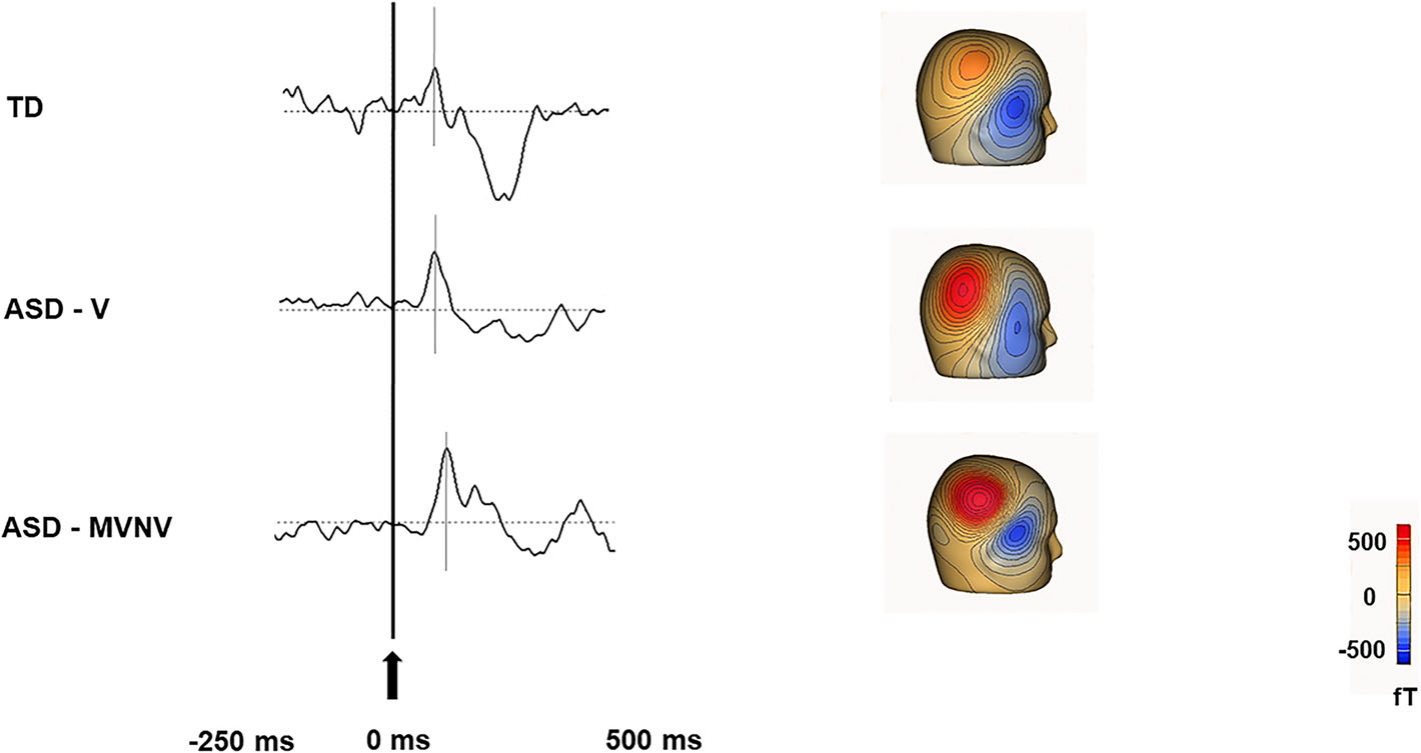
Source modeled activity waveform from right superior temporal gyrus for a representative participant in each group. Black vertical lines on the waveform and arrow indicate stimulus onset (0 ms). Gray lines indicate M50 peaks: for the representative TD marked at 71 ms, for the representative ASD-V child marked at 81 ms, and for the representative ASD-MVNV child marked at 98 ms. A clear prolongation of latency is observed in ASD-V, which is exacerbated in ASD-MVNV

All analyses were performed blind to the participant group. Epochs 100 ms pre-stimulus to 500 ms post-stimulus were defined from the continuous recording. To correct for eye blinks, a typical eye blink was manually identified in the raw data (including EOG) for each participant. The pattern search function in BESA Research 6.1 (BESA GmbH, Germany) scanned the raw data to identify other blinks and computed an eye-blink average. An eye blink was modeled by its first component topography from principal component analysis (PCA), typically accounting for more than 99% of the variance in the eye-blink average. In addition to eye-blink activity, a heartbeat average was obtained and heartbeat activity was modeled by the first two PCA component topographies of a heartbeat average, typically accounting for more than 85% of the variance in the heartbeat average. Scanning the eye blink and heartbeat-corrected raw data, epochs with other artifacts (not clearly identifiable as blinks or heartbeat) were rejected by amplitude and gradient criteria (amplitude > 300 fT, gradients > 25 fT/cm). Noncontaminated epochs were averaged, and a 1 Hz (12 dB/octave, zero-phase) to 55 Hz (48 dB/octave, zero-phase) band-pass filter was applied. Using all 275 channels of MEG data, determination of the strength and latency of M50 and M100 sources in the left and right STG was accomplished by applying a standard source model to transform each individual’s raw MEG surface activity into the brain space (MEG data co-registered to the Montreal Neurologic Institute (MNI) averaged brain) using a model with multiple sources [51–53]. In particular, the standard source model applied to each subject was constructed by including (1) left and right STG dipole sources (placed at left and right Heschl’s gyrus) and (2) nine fixed regional sources that modeled brain background activity and served as probe sources for additional activity. The eye-blink and heartbeat source vectors derived for each participant were also included in each participant’s source model to remove eye-blink and heartbeat activity [2, 24]. The final source model served as a source montage for the raw MEG [54, 55]. As such, the MEG sensor data was transformed from channel space into brain source space where the visualized waveforms were the modeled source activities. This spatial filter disentangled the source activities of the different brain regions that overlapped at the sensor level. Of note, although the latency of the 50 and 100 ms STG responses was obtained using a dipole source placed at a standard location, in each subject left- and right-hemisphere, dipoles were oriented at the maximum of the individual M50 and M100. A model goodness of fit requirement was set at > 80%. As such, orientation of the standard STG sources was optimized in each subject. Left and right M50 (50–125 ms) and M100 (100–250 ms) peaks were defined from the source waveform incorporating a prestimulus baseline period (− 100 ms to 0 ms) and paying close attention to magnetic field topography to ensure appropriate peak assignments (Fig. 1).

### Statistics

Potential effects of group (TD, ASD-V, ASD-MVNV) on age and clinical assessment data were evaluated with analysis of variance. Potential effects of group and hemisphere (LH, RH) on M50 and M100 latency and amplitude were evaluated with linear mixed models (LMMs) using these fixed effect factors and their interactions, subject as a random effect and age as a covariate. Hierarchical regression assessed the association of language ability and cognitive ability above and beyond effects of age and hemisphere on M50 and M100 latency and amplitude. Bonferroni corrections were applied for multiple comparisons. All statistical analyses were performed with SPSS Statistics Version 25 (IBM, Armonk, USA).

## Results

### Demographics

As shown in Table 1, 105 participants (8 to 12 years) were included in the final analysis (ASD-MVNV: *n* = 16, 9.85 ± 1.32 years; ASD-V: *n* = 55, 10.64 ± 1.31 years; TD: *n* = 34, 10.18 ± 1.36 years). Twenty-four participants (*n* = 14 ASD-MVNV; *n* = 9 ASD-V; *n* = 1 TD) had previously been eliminated as they either did not complete scans, did not complete or meet criteria on neuropsychological assessments, or did not have analyzable MEG (degraded by artifact or incomplete acquisition). There was no statistically significant main effect of group on age (*p* > 0.05). As expected, there were main effects of group on SCQ [*F* (2, 77) = 94.64, *p* = 0.001], with TD < ASD-V < ASD-MVNV and all pairwise post hoc *t* tests significant (ps = 0.001). Similarly, there was a significant effect of group on nonverbal IQ [*F* (2, 83) = 75.29, *p* = 0.001], with TD > ASD-V > ASD-MVNV and, again, all pairwise post hoc *t* tests significant (ps = 0.001). For the Vineland communication domain score, there was a significant main effect of group [*F* (2, 49) = 37.84, *p* = 0.001] with no difference between TD and ASD-V and with both significantly greater than ASD-MVNV (*p* = 0.001). For the *verbal* children, there were main effects of group (TD > ASD-V) on GAI/FSIQ [*F* (1, 62) = 13.50, *p* = 0.001] and verbal IQ [*F* (1, 68) = 12.97, *p* = 0.001].

**Table 1:** Characteristics of study participants

	Typically developing	ASD-verbal	ASD-minimally verbal/nonverbal
Number of participants	34	55	16
Gender(M:F)	29:5	45:10	13:3
Handedness (R:L:A)	26:8:0	45:9:1	10:4:2
Age	10.18 ± 1.36	10.64 ± 1.31	9.85 ± 1.32
Social Communication Questionnaire	2.62 ± 2.47	18.62 ± 6.56	26.13 ± 7.56
Communication skills (Vineland)	93.31 ± 13.13	93.21 ± 16.45	48.00 ± 17.62
Nonverbal IQ	112.83 ± 12.69	98.28 ± 16.58	55.73 ± 12.44
Full Scale IQ [estimated]	113.56 ± 14.09	98.22 ± 18.03	–

Communication skills: Communication Subscale from the Vineland-II/Vineland-III

Nonverbal IQ: nonverbal IQ score from the WISC-IV/WISC-V/Leiter-3; Nonverbal Spatial Composite from the DAS-II

Full Scale IQ [estimated]: General Ability Index or Estimated FSIQ score from the WISC-IV/WISC-V; General Conceptual Ability Score from the DAS-II; unavailable for ASD-MVNV group

### Trials

There was indeed a statistically significant main effect of group on evaluable trial count [TD = 477.15 ± 2.89; ASD-V = 459.22 ± 3.66; ASD-MVNV = 420.06 ± 13.00; *F* (2, 209) = 22.82, *p* = 0.001]. ASD-MVNV showed statistically significant lower number of acceptable trials compared to both ASD-V and TD (*p* = 0.002, *p* = 001, respectively). In all cases, a minimum of nearly 200 trials were evaluable [TD (range 417–520), ASD-V (range 344–507), and ASD-MVNV (range 189–516)] and in most cases many more (which is considerably greater than in most paradigms in the literature) as we chose to deliver a large number of stimuli, anticipating exactly this type of potential data loss. The key reason for excluding trials was head (or body) movement.

### M50 and M100 latencies

**Fig. 2:**
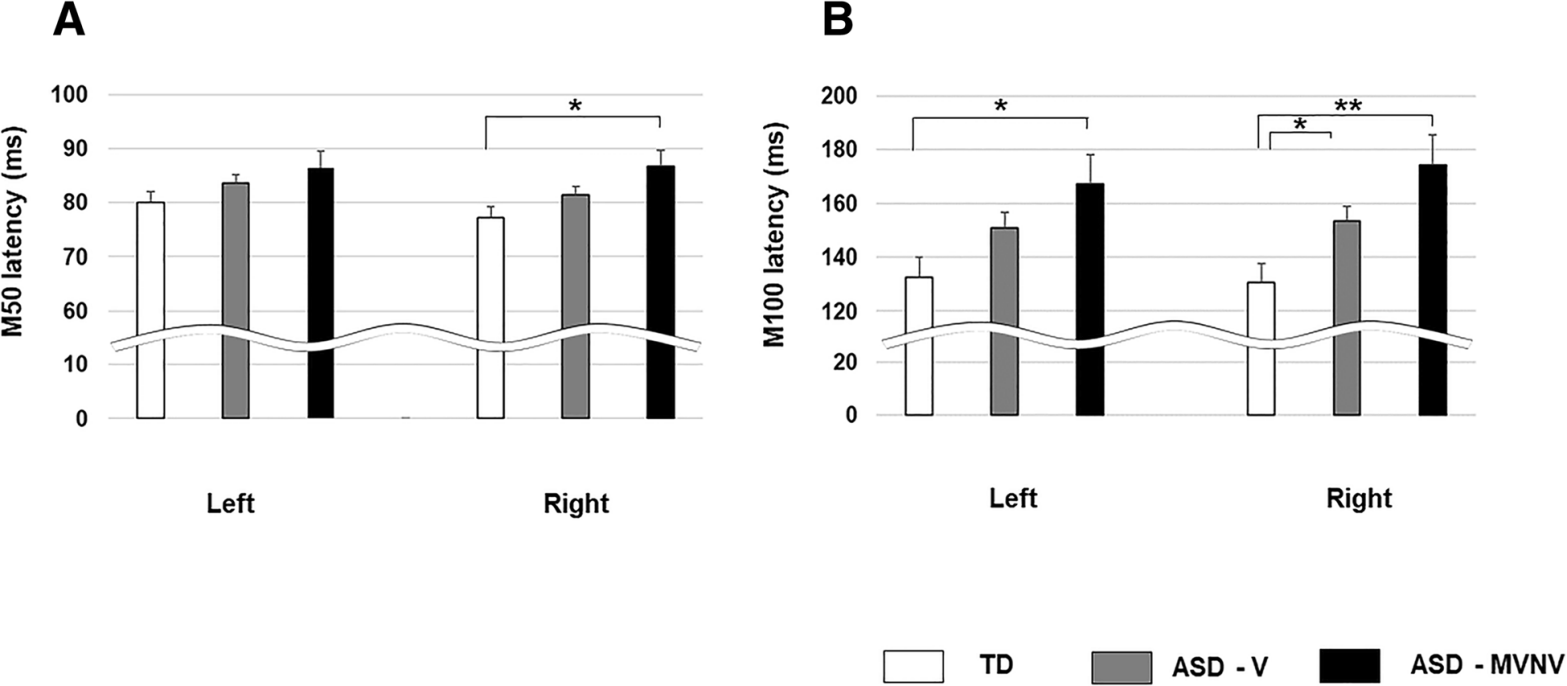
**a** Estimated marginal mean latencies by group across hemisphere for M50 latency. Error bars represent one standard error of the marginal means. There is a significant main effect of group on M50 latency (*p* < 0.05) across hemispheres. Post hoc tests indicated ASD-MVNV showed significantly delayed M50 latency compared to TD across hemispheres (*p* < 0.05), which was, however, driven by a significant delay in the right hemisphere (*p < 0.05) only (shown in the figure). **b** Estimated marginal mean latencies by group across hemisphere for M100 latency. Error bars represent one standard error of the marginal means. There is a significant main effect of group on M100 latency (*p* < 0.05). Post hoc tests indicated ASD-MVNV showed a delayed M100 latency compared to TD across both hemispheres (*p < 0.05, **p < 0.01), while ASD-V showed a near-significant tendency towards delayed M100 compared to TD (*p* = 0.07), which reached significance in the right hemisphere only (*p = 0.039) consistent with prior reports of right hemisphere bias in latency delay [42]

A linear mixed model (LMM) with fixed effects of group, hemisphere, and age (and with subject as a random effect) showed an effect of group on M50 latency [TD = 78.6 ± 1.8 ms; ASD-V = 82.5 ± 1.4 ms; ASD-MVNV = 86.6 ± 2.7 ms; *F* (2, 98.30) = 3.27, *p* = 0.042], with no effect of hemisphere [LH 83.4 ± 1.4 ms; RH 81.8 ± 1.4 ms; *F* (1, 96.67) = 0.613, *p* > 0.05] and no interactions [*p* > 0.05]. A pairwise post hoc test indicated ASD-MVNV showed significantly delayed M50 latency compared to TD across hemispheres, but this was driven by significant differences in the right hemisphere only (*p* = 0.047, Fig. 2a), while ASD-MVNV showed a non-significant trend towards delayed M50 latency versus ASD-V.

A similar LMM revealed a main effect of group on M100 latency [TD = 131.5 ± 7.1 ms; ASD-V = 152.2 ± 5.6 ms; ASD-MVNV = 170.8 ± 11.0 ms; *F* (2, 96.72) = 5.107, *p* = 0.008], with no effect of hemisphere [LH 150.0 ± 4.8 ms; RH 153.0 ± 4.8 ms; *F* (1, 92.917) = 3.0 *p* > 0.05] and no interactions [*p* > 0.05]. Post hoc tests indicated ASD-MVNV showed a delayed M100 latency compared to TD across hemispheres (*p* = 0.01), which remained significant for each hemisphere considered separately (LH, *p* <0.05; RH, *p* = 0.004) (Fig. 2b). Overall, ASD-V showed a trend towards delayed M100 latency compared with TD (*p* = 0.074), with post hoc *t* tests showing significant delays in ASD-V compared to TD in the right hemisphere (*p* = 0.039) consistent with prior reports [42] and similar to M50 findings. Similarly to the M50 results, although ASD-MVNV tended to show a later M100 latency compared to ASD-V, there were no significant ASD-MVNV vs ASD-V differences in either hemisphere.

Across the whole cohort, when entered first in the regression, age and hemisphere together accounted for 16.1% of the variance (*p* = 0.001). When entered second, NVIQ accounted for an additional significant 8.3% of the variance in M50 latency (*p* = 0.001), and when entered third, the Vineland Communication Domain Standard Score accounted for a further significant 3.2% of the variance in M50 latency (*p*<0.05). However, when the order of entry of NVIQ and Vineland Communication Domain Standard Score was reversed, whereas Vineland continued to account for a statistically significant 10.5% of the variance in M50 latency (*p* = 0.001), NVIQ no longer accounted for significant additional variance (0.9%, *p* > 0.05), suggesting that the association with NVIQ implied above was a proxy for communication ability and better indexed by the Vineland Communication Domain. Across the entire cohort, the two measures (Vineland Communication Domain and NVIQ) were correlated (*R*^2^ = 53.4%, *p* = 0.001). This pattern of findings held for the full cohort as well as the ASD sub-cohort (pooling ASD-MVNV and ASD-V).

When predicting M100 latency, across the whole cohort, when entered first in the regression, age and hemisphere together accounted for 4.8% of the variance (*p* = 0.029). When entered next (second), NVIQ accounted for a non-significant 2.4% of the variance in M100 latency (*p* > 0.10), and when entered third, the Vineland Communication Domain Standard Score accounted for an additional significant 4.6% of the variance in M100 latency (*p* = 0.046). When the order of entry was reversed, Vineland Communication continued to account for a statistically significant 6.9% of the variance in M100 latency (*p* = 0.006), and NVIQ was again not significant (0.1%, *p* > 0.05).

### M50 and M100 amplitude

A linear mixed model (LMM) with fixed effects of group, hemisphere, and age (and with subject as a random effect) showed an effect of group on M50 amplitude [TD = 3.58 ± 0.41 nAm; ASD-V = 3.93 ± 0.33 nAm; ASD-MVNV = 5.99 ± 0.64 nAm; *F* (2, 98.79) = 5.23, *p* = 0.007], with no effect of hemisphere [LH 4.33 ± 0.29 nAm; RH 4.67 ± 0.29 nAm; *F* (1, 98.06) = 0.09, *p* > 0.05] and no interactions [*p* > 0.05]. Pairwise post hoc tests indicated ASD-MVNV showed significantly increased M50 amplitudes compared to TD across hemispheres (*p* = 0.006). ASD-MVNV also showed significantly increased M50 amplitudes compared to ASD-V across hemisphere (*p* = 0.016). A LMM revealed no main effect of group on M100 amplitude [TD = 4.51 ± 0.57 nAm; ASD-V = 5.06 ± 0.46 nAm; ASD-MVNV = 6.46 ± 0.88 nAm; *F* (2, 99.01) = 1.72, *p* > 0.05], with no effect of hemisphere [LH 5.12 ± 0.43 nAm; RH 5.56 ± 0.43 nAm; *F* (1, 98.56) = 1.09, *p* > 0.05] and no interactions [*p* > 0.05].

Regarding associations between M50 amplitude, language, and communication ability, when entered after age, hemisphere had together accounted for 0.3% of the variance (*p* > 0.05), NVIQ accounted for an additional significant 21.3% of the variance in M50 amplitude (*p* = 0.001), Vineland Communication Domain Standard Score entered next did not account for significant additional variance in M50 amplitude (1.5%, *p* > 0.05). When the order of entry for NVIQ and Vineland was reversed, Vineland accounted for a significant 15% of the variance in M50 amplitude (*p* = 0.001) and NVIQ accounted for significant additional variance (7.2%, *p* = 0.003). When predicting M100 amplitude, when entered after age, hemisphere (together 2%, *p* > 0.05), NVIQ accounted for a further significant 7.6% of the variance in M100 amplitude (*p* = 0.005), and Vineland Communication Domain Standard Score entered next did not account for additional variance in M100 amplitude (3%, *p* > 0.05). When the order of entry for NVIQ and Vineland was reversed, Vineland did not account for significant variance in M100 amplitude (1.9%, *p* > 0.05), while NVIQ continued to account for significant additional variance (6.0%, *p* = 0.013).

## Discussion

Findings indicated delayed STG M50 and M100 neuromagnetic responses to simple auditory tones in MVNV children who have ASD compared with ASD-V children or TD children. Furthermore, longer latencies were associated with poorer communication skills as indexed by the Vineland Adaptive Behavior Scales Communication Domain. In the present study, M50 latencies were delayed by ~ 4 ms in ASD-MVNV compared to ASD-V children and ~ 8 ms in ASD-MVNV children compared to TD children. M100 latencies were delayed by ~ 18 ms in ASD-MVNV versus ASD-V children and ~ 39 ms in ASD-MVNV versus TD children. Findings were again consistent with reports using MEG with simple tones in verbal children with ASD but without intellectual disability [42, 45]. Successful recording of auditory evoked neuromagnetic fields in the ASD-MVNV cohort reflects the utility of the MEG-PLAN approach and indicates the possibility of evaluating relationships between electrophysiological responses and behavioral abilities across a broad range of language abilities.

The mechanism underlying the delayed latency of M50 and M100 responses in ASD-V children and the exacerbation of this delay in ASD-MVNV children is unclear. Reports using EEG have shown delayed latencies in individuals with intellectual disability, noting delayed N100 latencies to simple tones and vowels in children with Down syndrome aged 10–12 years compared with TD children, and with the authors speculating that the prolonged auditory latencies in individuals with Down syndrome might be associated with deficits in myelination or as a result of thyroid dysfunction [15]. Findings supporting maturational changes in auditory pathway white matter as influencing conduction velocity (and shorter M50/M100 latencies with increasing age) in typically developing children [41] were not, however, replicated in a cohort of children with ASD (whose M50/M100 responses to sinusoidal tones were nonetheless delayed), leading to the hypothesis that synaptic transmission (or other factors) may *also* influence auditory latency delay [45].

Regarding local synaptic transmission, development of layers (lower III to VI) in auditory cortex is known to occur between 6 months and age 5 years [36], with superficial layers (upper layer III and II) continuing to mature until about age 12 years [16, 29, 30]. Based on these findings, these researchers suggested that the auditory 50 ms response reflects recurrent activation in layers III and IV, the termination zone of thalamocortical pathways that are almost fully developed in by age 6 years. The 100 ms auditory response reflects activation of cortical layers upper III and II, areas not fully developed until age 12 years [37, 38]. In the present study, delayed M50 and M100 latencies were found in ASD-MVNV and ASD-V, with ASD-MVNV showing exacerbation of the latency delay. Present findings of latency delays in *both* M50 and M100 perhaps indicate that ASD-MVNV children have more severe abnormalities in maturation of the local neural circuits generating these responses compared with ASD-V children.

An alternative (or adjunct) to the cortical maturation hypothesis is consideration of the thalamus itself and thalamocortical connectivity. The thalamus is a complex brain structure through which nearly all sensory information is routed and thus plays a key role in sensory modalities [6] as well as cognitive domains ranging from language to social motivation [5]. Concerning thalamic abnormalities in individuals with ASD, reports have documented reduced thalamic volume [60] and abnormal thalamocortical networks involved in language processing [31] in children and adults. Linke et al. [23] also reported increased connectivity between the thalamus and auditory cortex in children and adolescents with ASD, with these abnormalities associated with reduced cognitive and behavioral abilities (such as social interaction and communication). Based on these findings, abnormalities in thalamocortical networks have been hypothesized to be related to language and communication impairment in MVNV children on the autism spectrum. Further studies are needed to more fully explore this hypothesis. Another factor supporting a connectivity hypothesis is the relative increase in M50 amplitude from TD to ASD-V to ASD-MVNV. One interpretation of this is decreased lateral inhibition of the M50 evoked response in ASD, with the degree of inhibition decrease scaling with symptom severity. Further studies are warranted to explore such hypotheses.

Another finding is the association between auditory evoked response latencies and language and communication skills (Vineland Communication Domain standard score). The M50 and M100 responses are primarily generated in the STG [27, 35, 61], and the STG has figured prominently in models of receptive language function and impairment [12]. Previously, Oram Cardy et al. [34] reported that right-hemisphere M50 latency was associated with language ability and also that right-hemisphere M50 latency differentiated language-impaired and non-language-impaired children who have ASD. In the present study, associations between M50/M100 latency and language ability were observed bilaterally across the three groups as well as when restricting analyses to only the children who have ASD.

Although the regressions suggested the relationship with language and communication as dominant, with the relationship with NVIQ attributable to the partial correlation between measures of language ability and NVIQ, a potential limitation of this study is the coupling of language and general cognitive abilities in many of the children in the MVNV cohort. Studies with groups differentiated by language ability, cognitive ability, and diagnosis are needed to fully disambiguate these associations between biological markers and behavioral/clinical measures (e.g., children who are minimally verbal but who have nonverbal abilities nearing the age-appropriate range OR children *with* intellectual disability but *without* ASD). A potential limitation of the findings is their unknown specificity—other developmental disorders may also share this neurophysiological observation. Interestingly, in a prior study in specific language impairment (SLI) [44], we observed neurotypical M100 latencies, but delayed MMF responses (which have also been reported in ASD-MVNV [28]). Nonetheless, although specificity would be required for a diagnostic marker (and these comparative pan-disorder studies should be pursued to determine exactly this degree of specificity), an overlapping phenotype is not without value as it points to pathways disrupted in common across disorders. This has the potential to lead to rational selection of therapeutic intervention (e.g., by repurposing pharmaceuticals). Also, if the index has prognostic value (scaling with degree of impairment), it may offer a quantitative index to monitor the efficacy of interventional strategies. Another study limitation is a focus only on MEG. Other brain imaging measures were not obtained (e.g., cortical myelin content, diffusion tensor imaging, or GABA magnetic resonance spectroscopy obtained via MRI/MRS) due to difficulties encountered when attempting to perform MRI in the ASD-MVNV population. Extensions of our MEG-PLAN behavioral/technical techniques [22] to accommodate MRI scanning in populations such as these are warranted. Furthermore, although we attempt to generate generalizable findings by including the often-excluded ASD-MVNV cohort, some residual bias must remain. A fraction of our ASD-MVNV cohort could not participate in the MEG scanning despite MEG-PLAN, and thus, we have potentially biased our inclusion to a select (possibly slightly higher-functioning) subset of ASD-MVNV. Nonetheless, we believe that including even a subset of children in the ASD-MVNV population offers a pathway towards more generalizable conclusions and represents an advance over prior studies which exclude based on cognitive impairment.

## Conclusion

The present study indicated delayed M50 and M100 responses in MVNV children who have ASD. Latency delays were greater in ASD-MVNV than ASD-V, and latency delays were associated with poorer language ability. Findings suggest that measures of auditory cortex neural activity are objective markers of auditory cortex dysfunction in ASD, with the association with language ability indicating that these measures have prognostic and, ultimately, potential treatment value.

## Data Availability

The datasets used and analyzed during the current study are available from the corresponding author on reasonable request.

## Abbreviations

ASD: Autism spectrum disorder
ADOS: Autism Diagnostic Observation Schedule
ADI-R: Autism Diagnostic Interview-Revised
DAS: Differential Ability Scale
EEG: Electroencephalography
HF: High functioning
Leiter: Leiter International Performance Scale
NVIQ: Nonverbal Intelligence Quotient
MEG: Magnetoencephalography
MNI: Montreal Neurologic Institute
MVNV: Minimally verbal/nonverbal
STG: Superior temporal gyrus
SCQ: Social Communication Questionnaire
TD: Typically developing
WISC: Wechsler Intelligence Scale
Vineland: The Vineland Adaptive Behavior Scale

## Footnotes

1 Individuals on the autism spectrum, their parents, and professionals in the field have unique and overlapping opinions regarding the use of person-first (e.g., children with ASD) or identity-first (e.g., autistic child) language [20]. With respect for divided opinions, we use both approaches to terminology in this paper.
